# Vision Loss as a Presenting Symptom of Vitamin B12 Deficiency

**DOI:** 10.7759/cureus.60113

**Published:** 2024-05-11

**Authors:** Izzati Othman, Evelyn Tai, Sangeeta Kuganasan, Norlelawati Abu

**Affiliations:** 1 Department of Ophthalmology, Hospital Tuanku Jaafar Seremban, Negeri Sembilan, MYS; 2 Department of Ophthalmology and Visual Science, Hospital Universiti Sains Malaysia, Kubang Kerian, MYS

**Keywords:** vitamin b12 deficiency, vitamin b supplementation, malnutrition risk, metabolic optic neuropathy, nutritional blindness

## Abstract

Nutritional optic neuropathy is a rare and often overlooked factor leading to bilateral, symmetrical, and gradual visual impairment. This condition falls within the category of metabolic neuropathies. We documented a case involving bilateral nutritional optic neuropathy attributed to pancytopenia associated with vitamin B12 deficiency. A healthy 65-year-old Indian woman reported a bilateral, progressive, painless decline in vision over the past six months. She had a history of reduced oral intake for the preceding year and denied experiencing any gastrointestinal or constitutional symptoms. Bilateral visual acuity was 1/60. Examination revealed pale optic discs with attenuated vessels in both eyes and a cup-disc ratio of 0.3. The blood analysis showed low indices and a deficiency in serum vitamin B12. Despite undergoing treatment, her vision remained impaired due to the chronic nature of the condition. This case highlights the importance of identifying visual symptoms in an elderly woman experiencing malnutrition caused by inadequate dietary habits, which leads to bilateral nutritional optic neuropathy.

## Introduction

Metabolic optic neuropathies are classified into three subcategories: heredodegenerative (e.g., Leber's hereditary optic neuropathy), nutritional deficiencies (e.g. vitamins B12 or folic acid), or toxicities (e.g. ethambutol or cyanide) [1]. Ophthalmic manifestation is an uncommon occurrence in vitamin B12 deficiency, which can manifest as presenting features or as one of the neurological complications associated with the deficiency [2]. It is characterized by painless, progressive, bilateral, and symmetrical reduction in visual acuity, often accompanied by color vision impairment [1,3]. Progression of the neuropathy is linked to optic nerve atrophy, resulting in total disc pallor, which may lead to irreversible optic neuropathy [3,4]. Management of nutritional neuropathy involves dietary supplementation to address the deficient nutrients [3,5]. This article details a case of bilateral optic neuropathy due to vitamin B12 deficiency with pancytopenia in an elderly individual affected by malnutrition. This case was presented orally during the 38th Asia Pacific Academy of Ophthalmology Congress meeting. The patient provided informed consent for the documentation of this case report.

## Case presentation

**Figure 1:**
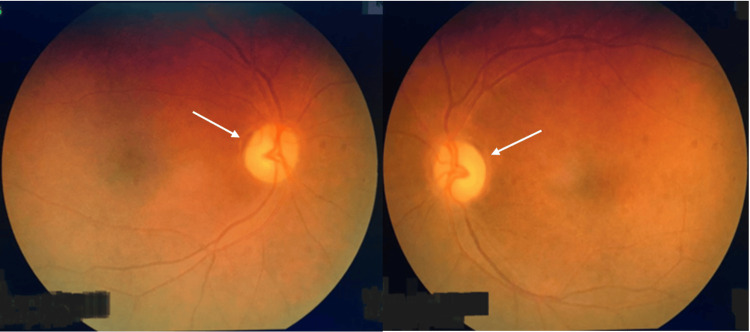
Bilateral fundus examination showed pale discs with narrowed vessels

A 65-year-old woman, previously in good health, experienced a gradual, painless decline in vision in both eyes over the past six months. She had experienced decreased appetite but did not report any gastrointestinal or constitutional symptoms for the past year. Her visual acuity was measured at 1/60 in both eyes. A bilateral fundus examination revealed pale discs with narrowed vessels and a cup-disc ratio of 0.3 (refer to Figure 1).

Apart from these ocular findings, her ocular and systemic examinations were unremarkable. Blood analysis revealed significantly low indices, including a haemoglobin level of 4.7 g/dL, a white blood cell count of 2.5 x 10^3^/μL, and a platelet count of 83 x 10^9^/L. Examination of the peripheral blood film indicated characteristics consistent with mixed nutritional deficiency anemia (Table 1).

**Table 1:** Peripheral blood film of haematological parameters Haemoglobin (HGB): Severe anemia with poor retic response; Red Blood Cells (RBC): Macrocytic red cells, oval macrocytes with few hypochromic red cells and some elliptocytes with tear drop cells seen; Platelet (PLT): Thrombocytopenia without platelet clumping seen, manual platelet count-10-15 high power field (hpf); White Blood Cells (WBC): Leukopenia with severe neutropenia, some neutrophils are hypersegmented but no blast/immature cells seen; Interpretation: Features suggestive of megaloblastic anemia (supported by low serum Vitamin B12 level: 72.22 pmol/L); HCT: Haematocrit test; MCV: Mean corpuscular volume;  MCH: Mean corpuscular hemoglobin; MCHC: Mean corpuscular haemoglobin concentration; RDW-CV: Red blood cell distribution width-corpuscular volume.

Parameter	Result	Reference Range / Unit
WBC	2.5	4.0 -10.0 x 10^9/L
RBC	1.33	3.80-4.80 x 10^12/L
HGB	47.0	120-150 g/L
HCT	0.136	0.360-0.460 L/L
MCV	102.3	83.0-101.0 f/L
MCH	35.3	27.0-32.0 pg
MCHC	346	315-345 g/L
PLT	83	150-400 x 10^9/L
RDW-CV	2.8	11.6-14.0 %
Neutrophils %	14.10	40.0-80.0 %
Immature granulocytes %	0.0	0.0-5.0 %
Lymphocytes %	81.9	20.0-40.0 %
Monocytes %	3.6	2.00-10.0 %
Eosinophil %	0.40	1.00-6.00 %
Basophil %	0.00	1.00-2.00 %
Neutrophil	0.35	2.00-7.00 x 10^3/L
Immature granulocytes	0.00	10^3/L
Lymphocytes	2.03	1.00-3.00 x 10^3/L
Monocytes	0.09	0.20-1.00 x 10^3/L
Eosinophil	0.01	0.02-0.50 x 10^3/L
Basophil	2.2	0.02-0.10 x 10^3/L
Reticulocytes %	2.2	0.5-2.5 %
Reticulocytes	29.0	50-100 x 10^9/L

Additionally, the serum vitamin B12 level was found to be low at 72.22 mmol/L (refer to Table 2).

**Table 2:** Chemical pathology laboratory report TIBC: Total iron-binding capacity. Serum Vitamin B12 level was low at 72.22 pmol/L.

Parameter	Result	Reference range / Unit
Serum Ferritin	313.30	10-291ng/mL
Serum Iron	24.60	9-30.4 umol/L
Serum TIBC	29.76	44.75-76.08 umol/L
Serum Folate	37.92	Deficient: 0.79 nmol/L, Intermediate: 7.64 nmol/L, Low: > 12.19 nmol/L
Serum Vitamin B12	72.22 (Deficient)	Normal:156-672 pmol/L, Deficient:24-181 pmol/L

**Figure 2:**
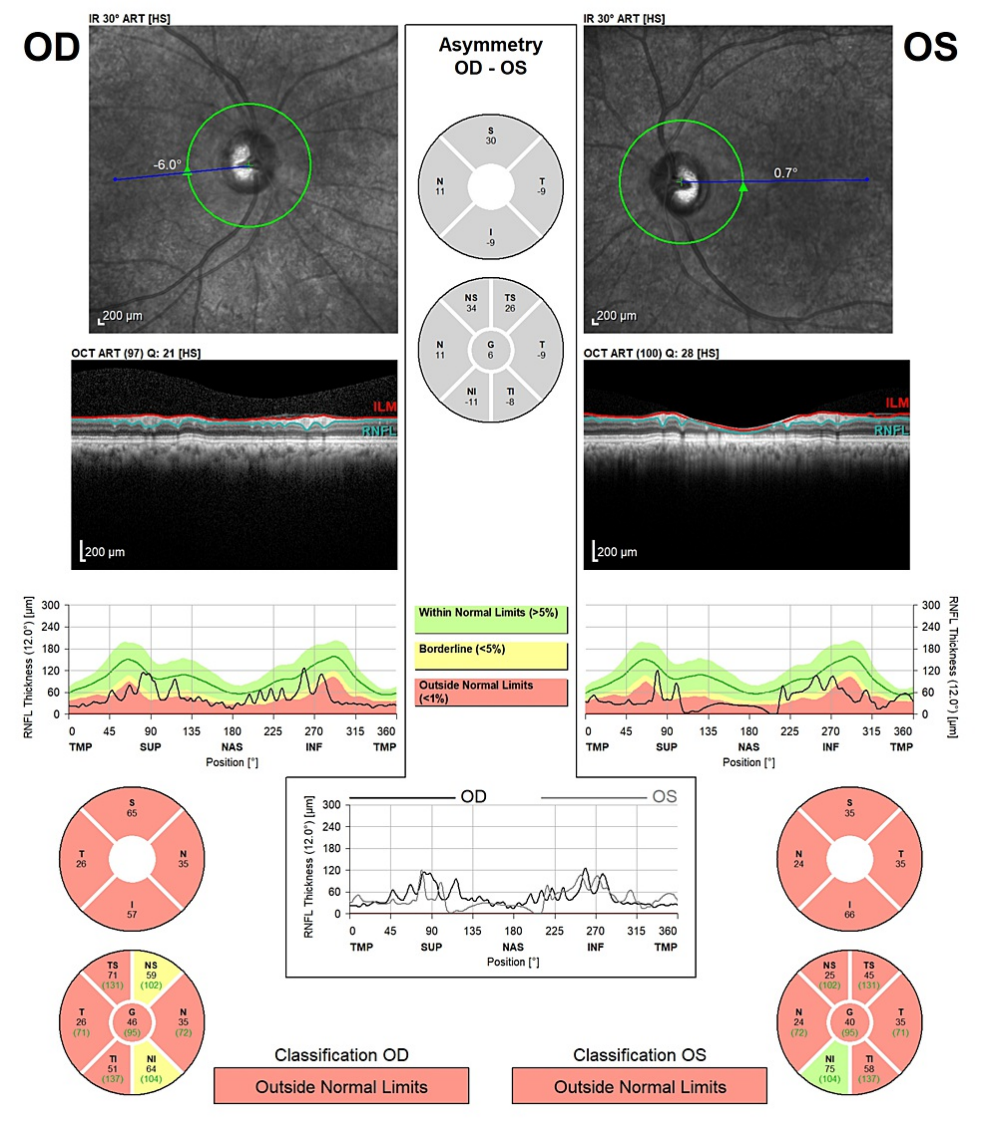
Optic nerve head optical coherence tomography image The tomography revealed thin retinal nerve fiber layers in all quadrants.

Optical coherence tomography showed thinning of the retinal nerve fiber layers in all quadrants (refer to Figure 2).

The anti-intrinsic factor antibody test yielded negative results. The diagnosis indicated bilateral nutritional optic neuropathy due to vitamin B12 deficiency with pancytopenia. Treatment involved the administration of intramuscular cyanocobalamin and oral hematinics. Despite the intervention, her vision remained impaired during her latest evaluation six months after the initial presentation, likely due to the chronic nature of her condition.

## Discussion

Nutritional optic neuropathy presents with bilateral, symmetrical visual impairment, centrocecal scotoma (which can be either unilateral or bilateral), dyschromatopsia, and a reduction in contrast sensitivity. This condition leads to a gradual, painless loss of vision, typically presenting as severe and symmetric [1,5]. The absence of a relative afferent pupillary defect is attributed to the symmetrical presentation of the condition [1,5]. 

Nutritional optic neuropathy arises from an inadequate dietary supply of essential nutrients crucial for the normal function of optic nerve fibers. The condition is frequently linked to deficiencies in folic acid and the vitamin B complex, often associated with malnutrition, suboptimal dietary practices, improperly implemented vegetarian diets, or prolonged alcohol abuse. Additionally, obese individuals following bariatric surgery form another at-risk group for nutritional optic neuropathy [3,5-9].

This condition is primarily attributed to a deficiency in B-complex vitamins, particularly thiamine (vitamin B1) and cyanocobalamin (vitamin B12). Additionally, deficiencies in riboflavin (vitamin B2), niacin (vitamin B3), pyridoxine (vitamin B6), and folic acid have also been implicated [6]. These B-complex vitamins serve as cofactors in numerous cellular metabolic and catabolic reactions. Their deficiencies result in the accumulation of toxic byproducts within cells. Vitamin B9 and B12 play crucial roles in a reaction that synthesizes purines using formic acid as a reagent. Consequently, deficiencies in these vitamins lead to formic acid accumulation, which hinders the electron transport chain and suppresses mitochondrial function, thereby interfering with ATP production [4]. ATP depletion contributes to nerve dysfunction and eventual nerve death, explaining the progressive and irreversible nature of this disease [2].

Hematological abnormalities, such as pancytopenia, may be linked to vitamin B12 deficiency [10,11]. The presence of macrocytic anemia and blood film observations showing hypersegmented neutrophils (more than five lobes) are indicative of vitamin B12 deficiency. However, the conclusive test is the assessment of serum cobalamin levels, with a result below 148 pmol/l being highly sensitive for confirming the diagnosis [11]. It is advisable to evaluate serum cobalamin and folate levels concurrently, considering the overlap in metabolic pathways [11].

The correlation between hematological and neurological features has not been specifically established. This means that individuals with neurological symptoms may not necessarily show hematological abnormalities, and vice versa. Since a diagnosis of vitamin B-12 deficiency typically relies on detecting macrocytic anemia rather than neurological symptoms, it is possible that many vitamin B-12 deficient patients go undiagnosed, putting them at risk of irreversible neurological complications [12].

The treatment is mostly based on the replacement of deficient vitamins, as well as the elimination of risk factors for neuropathy. Various methods of administering vitamin B12 exist, including orally, intranasally, sublingually, subcutaneously, or intramuscularly (IM). The duration of therapy varies depending on the underlying cause. In cases where the deficiency is irreversible, such as gastric bypass and pernicious anemia, lifelong vitamin supplementation is necessary. In reversible malabsorption conditions, such as inadequate intake due to poor diet, alcoholism, or drug-induced factors, short-term treatment is typically recommended. Oral administration is commonly effective for patients with reversible causes, typically starting at a dosage of 1000 μg per day for a minimum of one month. Following this initial period, the dosage may either be maintained at the same level for ongoing treatment or reduced to 100 to 500 μg daily for individuals with mild reversible deficiencies. Ideally, the parenteral formulation is given intramuscularly (IM), starting with a daily dosage of 1000 μg for one week, followed by a weekly dose of 1000 μg for one month. The maintenance dose for IM or deep subcutaneous (SC) injections is typically 1000 μg monthly, with duration varying depending on the underlying cause. Treatment for folic acid deficiency generally involves a dosage ranging from 1 to 5 mg per day. The treatment duration may range from three to six months until the underlying cause of the deficiency is resolved [13].

## Conclusions

Nutritional optic neuropathy presents as a rare and frequently unnoticed condition marked by bilateral, symmetrical, and progressively deteriorating vision. It arises from a deficiency in neurotrophic vitamins crucial for sustaining nervous system health, including the optic nerve. In an elderly individual experiencing malnutrition, painless and gradual vision loss could signify optic neuropathy due to nutritional deficits. Prompt recognition of visual symptoms and initiation of treatment are imperative to prevent irreversible optic nerve damage. It is essential to gather a thorough dietary history in all cases of optic neuropathy presentation, as timely nutritional supplementation can potentially reverse vision loss.
